# Acute Quetiapine Intoxication: Relationship Between Ingested Dose, Serum Concentration and Clinical Presentation—Structured Literature Review and Analysis

**DOI:** 10.3390/jox14040085

**Published:** 2024-10-18

**Authors:** Matej Dobravc Verbič, Iztok Grabnar, Florian Eyer, Miran Brvar

**Affiliations:** 1 Centre for Clinical Toxicology and Pharmacology, University Medical Centre Ljubljana, 1000 Ljubljana, Slovenia; miran.brvar@kclj.si; 2 The Department of Biopharmaceutics and Pharmacokinetics, Faculty of Pharmacy, University of Ljubljana, 1000 Ljubljana, Slovenia; iztok.grabnar@ffa.uni-lj.si; 3 Department of Clinical Toxicology, TUM University Hospital, Technical University of Munich, 81675 Munich, Germany; florian.eyer@tum.de; 4 Centre for Clinical Physiology, Faculty of Medicine, University of Ljubljana, 1000 Ljubljana, Slovenia

**Keywords:** intoxication, quetiapine, toxicokinetics, toxicodynamics

## Abstract

Over the past decade, quetiapine has become one of the most commonly used psychotropic drugs in acute intoxication events worldwide. A structured literature review and analysis were conducted to assess the relationship between the kinetic and dynamic profiles in acute quetiapine intoxication. The correlation between dose and peak serum concentration (c_max_) was determined using Pearson’s correlation coefficient. Binary logistic regression was used to evaluate dose and c_max_ as predictors of the most common clinical events, signs and symptoms. One hundred and thirty-four cases of acute quetiapine ingestion were included in the analysis, with a median ingested dose of 10 g and a median c_max_ of 4 mg/L. The typical half-life was estimated to be 16.5 h, significantly longer than at therapeutic doses. For the immediate-release formulation, a biphasic disposition could not be excluded. Dose and c_max_ demonstrated a weak but significant correlation (r = 0.256; N = 63; *p* = 0.043). Central nervous system depression and tachycardia were the most common clinical signs. Higher doses and concentrations increased the risk of severe intoxication and were good predictors of intubation, tachycardia, hypotension, QT_c_ prolongation and seizures, but not QRS prolongation, arrhythmia, heart block, hypokalaemia or acidosis. The thresholds for dose and c_max_ that increased the risk for individual signs and symptoms varied widely. However, doses > 3 g or c_max_ > 2 mg/L can be considered as alert levels that represent a high risk for severe clinical course of acute quetiapine intoxication.

## 1. Introduction

Quetiapine (QTP) is a dibenzothiazepine antipsychotic drug approved for the treatment of schizophrenia, bipolar disorder and major depressive disorder [1,2,3]. However, estimates from several Western countries have shown that 60–70% of patients receiving QTP use it for indications outside the registration [4,5,6,7,8,9]. In particular, low-dose QTP has demonstrated efficacy and tolerability in a broader range of psychiatric disorders and has become a common substitute for benzodiazepines in the treatment of anxiety and insomnia [3,5,7,8,10,11,12,13,14,15,16,17,18,19,20,21,22]. As patients treated with QTP frequently suffer from debilitating and chronic psychiatric conditions, intoxication events, including suicide attempts with QTP, are not uncommon [23]. Over the past decade, QTP has become one of the psychotropic drugs most commonly implicated in acute intoxication events worldwide [17,18,24,25].

The severity of QTP intoxication may be difficult to predict [12]. Reported toxicity varies widely across the dose range. Peak serum concentration may be a good predictor of QTP toxicity [2] but is infrequently measured [26]. The existing data from individual case reports and studies in this regard have been confounding [2,12,27,28,29,30]. With the lack of clinical trials related to a toxicological setting [31], determining the relationship between dose, concentration and clinical picture can provide essential information in the management of intoxications.

This review article focuses on the relationship between the kinetic and dynamic profiles in acute intoxication with QTP. In the first part, the existing knowledge is described. In the second part, previously published cases are collected from the literature, and the joint data are statistically analysed to determine the relationship between the ingested QTP dose, serum concentrations and individual toxicodynamic parameters.

## 2. Review of the Existing Literature

### 2.1. Quetiapine in Therapeutic Use

#### 2.1.1. Pharmacokinetic Profile and Dose–Concentration Correlation in Therapeutic Use

QTP is therapeutically administered in single or multiple oral doses, ranging from 25 to 800 mg daily. It is available in immediate-release (IR) and extended-release (XR) oral dosage forms [32]. In the therapeutic dose range, QTP exhibits linear pharmacokinetics [2,33,34,35,36,37,38]. Table 1 presents the kinetic parameters for the IR and XR formulations in therapeutic and toxic dose ranges.

At therapeutic doses, the time to peak concentration (t_max_) with XR form is delayed by approximately 3 h, and serum concentration peak (c_max_) is attenuated [19]. Release occurs over 20 h [51]. A similar relative exposure is achieved with both forms in the therapeutic range [41,51]. The steady-state c_max_ and area under curve (AUC) of 300 mg XR once daily and 150 mg IR twice daily are considered equivalent [51]. QTP population pharmacokinetic models were one compartmental with first-order absorption [42,45,46]. Interindividual variability of pharmacokinetic parameters was estimated to be 78% to 140% [42].

QTP is a CYP3A4 substrate and may interact with analgesic, anticonvulsant, antiarrhythmic and antiparkinsonian drugs [17]. Coadministration of ketoconazole, a potent CYP3A4 inhibitor, results in a 3–4-fold increase in QTP c_max_, whereas phenytoin and carbamazepine as CYP3A4 inducers cause a 5-fold increase in QTP metabolism and a similar decrease in steady-state c_max_ [34,57,59].

In the «Arbeitsgemeinschaft für Neuropsychopharmakologie und Pharmakopsychiatrie» (AGNP) consensus guidelines, the reference range for QTP serum/plasma concentrations are set at 0.1–0.5 mg/L [33,51,60,61,62]. However, the required concentrations vary for different mental conditions. QTP trough levels of 0.02–0.3 mg/L have been associated with efficacy [28], while concentrations of up to 1 mg/L have been described as therapeutic [23,33,62,63].

The relationship between QTP dose and concentration demonstrates large interindividual variability. In 62 patients treated with 37.5–1200 mg QTP daily, the concentration/dose (C/D) ratio varied 238-fold [64]. In a therapeutic drug monitoring (TDM) analysis of adult patients treated with 12.5–2600 mg QTP daily, C/D ratios ranged from 0.01 to 6.9 nmol/L/mg [65]. In certain subpopulations, the C/D correlation may be stronger. A linear relationship between QTP plasma levels and dose per kg body weight was found in 41 patients with schizophrenia, drug-induced psychosis or borderline personality disorder [66]. C/D correlation was strong in 12 elderly patients with selected psychotic disorders [67]. A weak positive linear C/D relationship was found in 180 children and adolescents with psychotic and mood disorders [68].

#### 2.1.2. Clinical and Adverse Effects of Quetiapine

QTP acts via several pathways in the central nervous system (CNS). The desired effects and the adverse drug reaction (ADR) profile follow receptor binding affinity, which is largely dose-dependent [14]. Sedative effects at low doses are mainly mediated by histaminergic (H_1_) and adrenergic receptors [1,2,28,37,69]. H_1_ antagonism is associated with increased appetite and weight gain [70]. α_1_ blockade can cause orthostasis, hypotension and reflex tachycardia [70]. At higher doses (>300 mg), the mood-stabilising and antipsychotic effects increase due to antagonistic effects on serotonin (5-HT_2A_, 5-HT_1A_, 5-HT_2C_) and dopamine (D_1_, D_2_) receptors [14]. Extrapyramidal symptoms do not normally occur at therapeutic doses due to lower affinity for D_2_ receptors [1,61]. Antimuscarinic ADRs, e.g., dry mouth or constipation, can be observed at higher doses [2]. Less common ADRs include dyspepsia, hyperprolactinaemia and sexual dysfunction [39,62,68,71,72,73,74,75]. As with other antipsychotics, QT_c_ prolongation is likely to be dose-related [76,77,78,79]. Other rare but serious ADRs include neuroleptic malignant syndrome (NMS), hepatotoxicity, myocarditis and blood dyscrasia [80,81,82,83,84,85,86,87,88]. The XR form is generally better tolerated and causes less drowsiness in the initial hours post-ingestion. However, its effects may last longer [26,39].

Certain ADRs, e.g., anticholinergic delirium, urinary retention, sinus tachycardia and increased intraocular pressure, can be partly attributed to one of the two active metabolites, norquetiapine (N-desalkylquetiapine, norQTP) [70,89,90,91].

#### 2.1.3. Relationship Between Dose or Concentration and Clinical Response

Data on the relationship between QTP dose or plasma concentration and clinical response are limited [61]. Most studies have failed to demonstrate a significant correlation [46,51,68,73,92,93,94,95,96]. In Chinese patients with bipolar disorder, QTP serum concentrations but not daily doses were strongly correlated with clinical outcomes [62]. In 41 patients with schizophrenia, drug-induced psychosis or borderline personality disorder, patient improvement correlated with plasma levels and dose/kg body weight within each diagnostic category but not between categories [66].

### 2.2. Quetiapine in Acute Intoxication

#### 2.2.1. Acute Toxicity Dose and Concentration Range

A single dose of QTP that presents an acute intoxication is not defined, and there is a large overlap with therapeutic doses. For atypical antipsychotics, an expert consensus panel suggested that ingestion of <5 times the initial adult single dose (i.e., <125 mg for QTP) presented a relatively low risk of severe intoxication in naïve patients ≥ 12 years old. For patients receiving chronic therapy with QTP, the suggested threshold was five times their personal single dose [97]. In a retrospective analysis, QTP intoxication was defined as “a one-time ingestion of more than 400 mg of QTP” [10]. On the other hand, in studies assessing the correlation between QTP plasma concentration and therapeutic response, daily doses of up to 1800 mg were used [51,98]. In a small open-label study, doses of up to 1600 mg daily were well tolerated during a four-week acute phase, and doses up to 1000 mg were used and tolerated in the maintenance phase [99].

In the AGNP guidelines, QTP plasma concentration > 1.0 mg/L is considered toxic. This threshold was calculated by doubling the upper limit of therapeutic concentration [21], which frequently defines the laboratory alert level for antipsychotics [100]. Based on case reports, a higher toxicity threshold of 1.8 mg/L has been proposed. Concentrations ≥ 1.9 mg/L have been reported as potentially comatose-fatal [101]. In postmortem analyses, blood levels > 1 mg/L generally indicated QTP as a contributing factor to death [69].

#### 2.2.2. Toxicokinetic Profile

The available toxicokinetic data for QTP are based on cohort studies and case reports (Table 1). The bioavailability in acute intoxication is lower than at therapeutic doses [40]. In part, this may be due to the anticholinergic effects of QTP [40]. The use of decontamination procedures, e.g., activated charcoal, when administered promptly, may significantly reduce the amount absorbed [35,40]. Gastric emptying may be prolonged due to acute stress associated with severe poisoning and coingested drugs [28,35,100,102]. In several cases of QTP IR ingestion, the t_max_ was >12 h [27,54]. The reported half-life (t_1/2_) has a wide range. Both linear and non-linear elimination have been suggested [35,40,54]. Clinical studies conducted during product registration in which therapeutic doses were analysed may underestimate t_1/2_ at toxic doses [33,40].

Pharmacobezoars may contribute to delayed absorption and t_max_, particularly with XR formulations, but have also been found in rare cases with IR formulations at doses > 2.5 g [54,100,103,104,105,106]. In an in vitro study of QTP XR, the bezoars remained stable for more than 4 h, and the drug release was reduced by 53% [107]. In addition to the delayed serum concentration increase, bezoars pose a risk of additional complications, e.g., gastric or intestinal obstruction [107].

#### 2.2.3. Toxicodynamic Profile

QTP demonstrates a variable range of toxicity [23,58]. Intoxication causes symptoms such as CNS depression, lethargy, confusion, drowsiness, tachycardia, hypotension, respiratory depression, anticholinergic symptoms and ataxia [1,2,10,12,26,33,58,97,108,109,110,111,112,113]. Orthostatic hypotension, miosis, agitation and delirium are commonly encountered [2,29,108,114]. Hyperglycaemia and hypokalaemia occur less frequently [52]. Severe toxicity may be more common than with other atypical antipsychotics, occurring in about one-sixth of patients [1,111,115]. Clinical signs include seizures, status epilepticus, rhabdomyolysis, respiratory depression, hypotension, coma and death [1,2,29,116,117,118,119]. In a review of 945 cases of QTP overdose, 388 cases (41.1%) required admission to the intensive care unit (ICU) [1]. Coma was reported in 10% of QTP intoxication cases [60]. Fatal outcomes are rare, and there are known cases of patients surviving a very high overdose [26,58]. On the other hand, deaths have been reported even after therapeutic doses [29]. The mortality risk is higher than with other atypical antipsychotics [120,121]. In acute poisoning, complications such as aspiration pneumonia can contribute to death [60]. An Australian analysis estimated a 1.2% death rate directly attributable to QTP overdose in cases with QTP detected in postmortem blood [115,122].

Sinus tachycardia occurs in the majority of patients after severe intoxication [106,123]. It results from antimuscarinic activity and may also be a reflex to α_1_-induced vasodilation and hypotension [2,52,58,103,124]. Hypotension is more common than with most other antipsychotics and occurs in up to 40% of patients [28,106,115,123]. In most cases, it can be resolved with intravenous fluid replacement. Profound hypotension resistant to volume resuscitation is rare [125]. CNS and respiratory depression are related to the antagonistic action on 5-HT and dopamine receptors [126,127]. The antimuscarinic effects can cause transient sedation [128]. Histaminergic sedation is predominant in lower dose ranges [14].

There are several published reports of QT_c_ interval prolongation [33,52,128], but its clinical significance remains controversial [1,129,130]. In a US multicentre cohort study, QTP caused severe QT prolongation, defined as QT_c_ ≥ 500 ms using Bazett’s correction, in 96 of 471 intoxication cases [131]. In a case series of QTP poisoning published by Balit et al., the uncorrected QT interval was not altered despite the QT_c_ increase. The authors argued that the prolonged QT_c_ interval resulted from overcorrection by Bazett’s formula due to tachycardia rather than from intrinsic cardiac toxicity [2]. Cardiac arrhythmias are very rare [107,132,133]. Ngo et al. reported two cases of ventricular tachycardia without torsade de pointes [1]. A single case report describes Brugada syndrome following QTP intoxication [134]. Widened QRS complexes can be a sign of immediate circulatory collapse [135]. They have been associated with an increased risk of seizures and ventricular dysrhythmia [136]. Heart block, possibly related to hypokalaemia, is not uncommon [27,137].

Seizures can be induced by antagonism on dopamine (D_2_), histamine or adrenergic receptors, as well as hypoxia [138]. They occur infrequently, typically within 4–8 h post-ingestion, and are self-limiting in the majority of cases [2,106,138]. In a retrospective 5-year case series, the incidence of seizures in acute QTP intoxication events was 2% [1]. Late-onset seizures (≥24 h post-ingestion) have been described after ingestion of QTP at doses of 4 g to 30 g [58,139,140]. The relationship between severity and QTP serum concentration has not been clarified, and seizures have been reported at therapeutic doses [141,142,143,144]. Similarly, myoclonus has been reported as a leading symptom of QTP intoxication [145] but may also occur at higher therapeutic doses administered therapeutically [146,147].

Agitation has been reported at higher therapeutic doses [148]. In acute intoxication, it may follow initial drowsiness and lethargy after several hours [149]. Anticholinergic delirium may also occur at a late stage after a period of decreased consciousness and persist for several days, possibly due to delayed QTP absorption and/or elimination [2,55,91]. Coingested drugs, clinical interventions (e.g., intubation), somatic complications and underlying psychiatric disorders may contribute to delirium [91,150,151].

Rhabdomyolysis has been described after a period of immobility of at least 14 h [152,153,154]. However, in the case of a 48-year-old man ingesting 12 g of QTP, rhabdomyolysis may have occurred due to a direct toxic effect of QTP, as no muscle wasting or pain was present [152]. NMS has been rarely reported and is considered idiosyncratic. It has occurred even at therapeutic doses as low as 12.5 mg daily [155,156,157,158,159,160,161]. It is characterised by fever, rigidity, autonomic instability and altered mental status. Elevated creatine kinase levels and leucocytosis are common. Without early recognition, it can lead to rhabdomyolysis, respiratory failure, acute kidney injury, seizures and death [162]. Following intoxication with the XR form, delayed toxicity with prolonged coma, rhabdomyolysis and aspiration pneumonia are more likely [26,32,40].

Treatment of acute QTP intoxication is symptomatic, and patients should be monitored [26,33]. There is no specific antidote. The use of activated charcoal within 6 h post-ingestion reduces the absorption of QTP by 35% and shortens the apparent t_1/2_ [33,35,163]. Gastric lavage may be considered for severe intoxications, ideally within 1 h post-ingestion [33]. Whole bowel irrigation has also been used [149]. During gastric lavage, a temporary increase in serum concentrations may occur [164]. QTP is not dialysable [85,165].

All patients experiencing symptoms beyond mild drowsiness should be referred to the Emergency Department, regardless of the ingested dose [97]. With an ingested dose < 3 g and a Glasgow Coma Scale (GCS) score of 15 for 4 h post-ingestion, patients may be discharged to a psychiatric unit [2]. The expected time of onset and peak of toxicity symptoms is within 6 h post-ingestion for the IR formulation [97]. For the XR formulation, the initial assessment of vital signs and mental status may not predict the severity of intoxication as the peak toxicity takes longer to develop [10]. If no sedation or tachycardia occurs within 12 h after an overdose with the XR formulation, severe intoxication can be excluded [10].

#### 2.2.4. Relationship Between Dose or Concentration and Clinical Presentation in Quetiapine Intoxication

While the clinical signs and symptoms vary across the QTP dose range, c_max_ may be a good predictor of toxicity [2]. However, despite its therapeutic implications, plasma concentration is not commonly measured in toxicology, and obtaining a measurement at t_max_ is challenging [26]. An unreliable medical history, coingested drugs and the use of decontamination procedures can complicate the correlation assessment [28]. Therefore, dose, serum concentration and clinical presentation generally demonstrated a poor correlation [12,27,28,29,30]. However, in a retrospective analysis of 18 cases, QTP dose correlated with both c_max_ and the severity of the outcome [2]. In a cohort study of 286 QTP intoxication events, there was a modest correlation between dose and minimum blood pressure, and dose influenced the probability of intubation [111].

In postmortem analyses, whole blood concentrations may be more informative than serum concentrations [166]. The concentration ratio between whole blood and serum exceeded 1 at concentrations > 2.5 mg/L [166], in contrast to therapeutic doses with a ratio of ~0.7 [167,168]. QTP is likely to undergo a significant postmortem redistribution from striated muscle, connective and adipose tissue [21]. Drug diffusion from bezoars in the gastrointestinal tract to adjacent tissues may occur following ingestion of large quantities [21]. In fatal QTP monointoxications, postmortem whole blood concentrations were above 7.2 mg/L [47,166,169]. In a study of 21 fatal cases, the authors reported the (central and peripheral) blood concentrations < 1 mg/L as “therapeutic”, i.e., not related to death, and concentrations > 1 mg/L as “above therapeutic” and death-related [53]. Higher postmortem blood concentrations, reaching up to 170 mg/L, were found in heart or central body cavity blood samples, probably due to a higher susceptibility for postmortem redistribution [21,47,169,170,171,172].

## 3. Analysis of Published Cases of Acute Quetiapine Intoxication

The aim of the analysis was to investigate the relationship between the ingested QTP dose, serum concentrations and individual toxicodynamic parameters with the joint data of published cases from the literature. While the clinical signs and symptoms of QTP intoxication have been well described, it remains unclear how specific parameters correlate with the ingested dose and concentration. Therefore, dose and concentration ranges for each dynamic parameter were investigated. Potential thresholds that represent an increased risk for the occurrence of individual signs and symptoms were determined.

### 3.1. Methods

#### 3.1.1. Literature Search and Inclusion/Exclusion Criteria

A literature search was conducted for relevant sources on QTP poisoning in the PubMed^®^ and EBSCO databases (including the Science Citation Index expanded). The search string was any combination of the words “quetiapine” or “Seroquel” and “poisoning”, “overdose” or “intoxication”. In the second phase, the references and the citing literature (if available on the website) were analysed. Book chapters, preclinical studies and in vitro studies were not analysed.

Cases of QTP intoxication in patients ≥ 12 years old were included if the ingested dose and/or serum/plasma concentration of QTP were reported, along with a description of the clinical presentation. Cases with coingested agents were included if QTP was described as relevant to the clinical course. Cases of ADRs and drug interactions with therapeutic doses of QTP were excluded. Doses representing acute intoxication were the ingestions of >5 times the initial adult single dose of QTP in naïve patients [97] or doses exceeding the approved therapeutic range, i.e., >800 mg, in patients with regularly prescribed QTP [173].

Postmortem cases were included if any information on the clinical picture before death was available, together with the postmortem QTP concentration or the ingested dose. Only concentrations from peripheral blood samples were captured. Heart or cavity blood concentrations were not included as values may have been exaggerated due to postmortem redistribution [69,171,174].

#### 3.1.2. Data Gathering and Statistical Analysis

The following data were extracted from the references: patient demographics (age, sex), details of QTP ingestion (amount ingested [mg], IR/XR form), QTP serum/plasma concentrations [mg/L], time from QTP ingestion and/or time from hospital admission to sampling [h], use of clinically relevant coingestants, treatment strategies and interventions (decontamination procedures, intubation, use of vasopressors, specific therapies), length of hospital stay [days], ICU admission, length of ICU stay [days], clinical events, signs and symptoms (lowest GCS score, tachycardia [heart rate ≥ 100 beats per minute], hypotension [blood pressure < 90/60 mm Hg, or <100/50 mm Hg, or explicitly stated], QT_c_ prolongation [QT_c_ > 480 ms, or >55 ms above baseline, or explicitly stated QT or QT_c_ prolongation], QRS prolongation [QRS > 100 ms], arrhythmia, heart block, seizures, agitation, delirium, acidosis [serum pH < 7.34], rhabdomyolysis [creatine kinase levels > 1000 U/L], hypokalaemia [serum potassium level < 3.5 mmol/L, or explicitly stated], hyperglycaemia [serum glucose level ≥ 9 mmol/L]. Based on the lowest GCS score, cases were categorised into four grades according to the severity of intoxication: Grade 1: death; Grade 2: GCS = 3–8; Grade 3: GCS = 9–14; Grade 4: GCS = 15. If the GCS score was not explicitly stated, a three-member panel reviewed the case to determine whether the GCS grade (1–4) could be estimated from the clinical description.

Coingestants, considered clinically relevant, were all drugs taken in the context of acute intoxication affecting CNS, i.e., drugs of abuse, ethanol, drugs of the Anatomical Therapeutic Chemical Classification Group N (nervous system drugs), baclofen or diphenhydramine. Other clinically relevant coingestants were drugs with suspected clinically relevant kinetic interaction with QTP, e.g., potent CYP3A4 inhibitors.

Cases with reported ingested QTP dose and concentration, together with the estimated time from ingestion to sampling, were included in the concentration–time analysis. All concentration time points were dose normalised to 10 g of QTP. Time points < 5 h post-ingestion for the XR QTP formulation were excluded from the t_1/2_ estimation.

Pearson’s correlation coefficient was used to determine the correlation between QTP dose and c_max_. Binary logistic regression was used to evaluate QTP dose and c_max_ as continuous predictor variables for individual toxicodynamic parameters (clinical events, signs and symptoms, and treatment strategies). For each parameter, cases were divided into two separate groups, the “event” group and the “no event” group. Logistic regression was performed for the parameters with at least 15 cases in the “event” group with reported QTP dose or concentration. The case distribution for each dynamic parameter was reviewed. Based on the distribution figures, additional logistic regression models with dose or concentration as categorical variables were created where appropriate. Pearson’s chi-square test was used to test the association between tachycardia and QT_c_ prolongation, and Fisher’s exact test was used to test the association between QT_c_ prolongation and arrhythmia. For all tests, a *p*-value < 0.05 was the threshold for statistical significance.

Data analyses were performed in Microsoft Excel 2016 (descriptive statistics, dose-normalised concentration–time figures) and IBM SPSS Statistics 25 (distribution figures, logistic regression and other statistical tests).

### 3.2. Results and Discussion

The literature search, conducted on 11 March 2024, yielded 5437 results in the EBSCO database and 508 results in PubMed. Relevant case reports, case series, clinical studies, review articles, conference abstracts or annual Poison Control Centre reports were extracted. A total of 444 QTP intoxication cases were closely reviewed. Finally, 63 cases were included in the dose–concentration correlation assessment, 58 cases in the dose-normalised concentration–time figures, and 134 cases in the toxicokinetic–toxicodynamic correlation analysis, which were collected from 68 case reports, 16 case series, two cohort studies and six annual Poison Control Centre reports [1,2,12,27,28,29,33,40,49,52,54,55,56,58,71,72,85,103,104,106,111,114,116,125,126,128,137,138,139,141,144,150,152,153,154,157,163,164,175,176,177,178,179,180,181,182,183,184,185,186,187,188,189,190,191,192,193,194,195,196,197,198,199,200,201,202,203,204,205,206,207,208,209,210,211,212,213,214,215,216,217,218,219,220,221,222,223,224,225,226,227,228]. For 20 cases from an original cohort study, additional primary data were provided by the authors [27].

#### 3.2.1. Dose–Concentration Correlation

The ingested QTP dose and the measured c_max_ demonstrated a weak but significant correlation using Pearson’s correlation coefficient (r = 0.256; N = 63; *p* = 0.043). Since large discrepancies were observed in several cases, it is questionable whether the measured concentrations in these cases really corresponded to the c_max_. In addition, the information on QTP dose and the time of ingestion may have been inaccurate. If only the cases with a reliable medical history were analysed, the correlation would probably be stronger. Uncertainties related to dose and the time of ingestion have previously been used to improve the stability of a predictive model [35]. However, a relatively weak correlation between dose and exposure appears to be inherent to QTP, as evidenced by population pharmacokinetic analyses, demonstrating large interindividual variability even at therapeutic doses [42]. Therefore, the measurement of QTP concentration is advised to predict the toxicodynamic profile.

**Figure 1:**
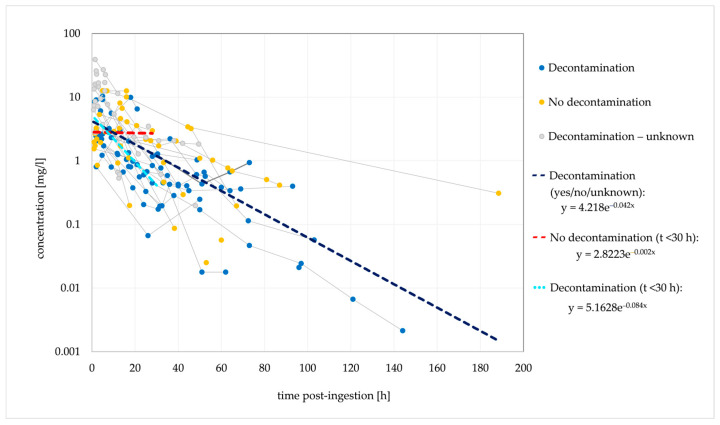
Observed dose-normalised quetiapine concentration vs. time (decontamination/no decontamination). Dashed lines are fitted monoexponential curves.

Of the 58 cases with an estimated time between ingestion and concentration measurements, 32 had multiple concentration measurements performed. One hundred and sixty-three concentration points are included in the dose-normalised concentration–time analysis (Figure 1).

**Figure 2:**
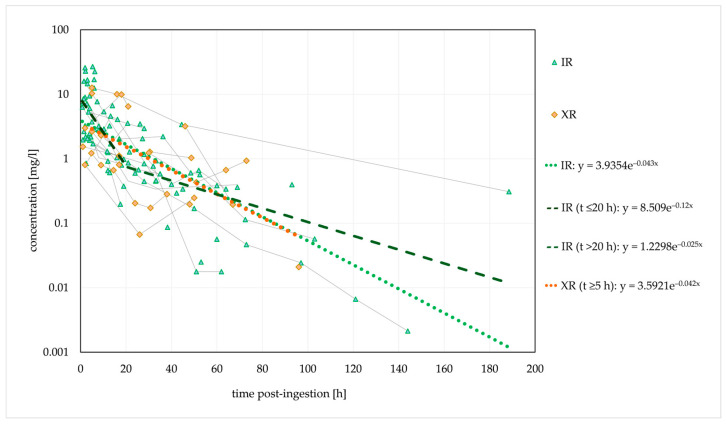
Observed dose-normalised quetiapine concentration versus time (immediate-release/extended-release formulation). Dashed lines are fitted monoexponential curves.

Based on the elimination rate constant (k_e_) of 0.042/h, the typical t_1/2_ was 16.5 h. In the cases where no decontamination was performed, a stagnant concentration trend line was observed for 30 h post-ingestion, suggesting that the absence of decontamination causes a delayed t_max_ and a delayed (or extended) concentration peak. Figure 2 shows the ingestions of IR and XR formulations separately; cases with unknown formulations are not included.

The estimated t_1/2_ was similar for both formulations. However, a biphasic disposition cannot be excluded for the IR form, with an initial t_1/2_ of 5.8 h (within 20 h post-ingestion, probably with a significant influence of the distribution) and a longer terminal t_1/2_ of 27.7 h (>20 h post-ingestion). Thus, the estimated initial t_1/2_ corresponded to the therapeutic t_1/2_.

A median t_1/2_ of 6.6 h was previously reported in a clinical evaluation of the effect of activated charcoal in 54 intoxication events [35]. The authors developed a one-compartment model with first-order absorption and first-order elimination that described the observed data. However, other studies and case reports demonstrated conflicting findings, and biphasic elimination was suggested in several cases [27,40,54,106]. This could indicate the presence of a third compartment, bezoar formation or hepatic redistribution [33,85,106]. Saturated hepatic metabolism is not very likely. The reported c_max_ values are several times lower than the previously estimated in vitro K_m_ values for the formation of QTP metabolites [57,106]. QTP was detected in plasma for a maximum of 7 days post-ingestion [47]. In the terminal phase, the rate of decline in QTP concentrations may be significantly reduced due to redistribution from a third compartment or the release of QTP from bezoars in the gastrointestinal tract. A slower elimination rate in the terminal phase could explain the longer duration of clinical symptoms in several described cases [54,106].

A second concentration peak was noted in 12 cases, five of which were intoxications with the XR form; in an additional four cases, the formulation was not explicitly stated. Activated charcoal was used in three cases, and gastric lavage was performed in five cases. Pharmacobezoar formation was recognised in three cases [164,204]. One case was a probable drug interaction with antiretroviral therapy [56]. The second concentration peak occurred between 20 and 56 h post-ingestion.

In the case of a 39-year-old female patient who had ingested 30 g of QTP (formulation unknown), norQTP concentrations were measured. The c_max_ of 10.2 µmol/L was reached 62 h post-admission, and the concentrations were still elevated after 80 h. The possibility of pharmacobezoar formation was not discussed [54,228]. Vignali et al. found norQTP in significant concentrations in 13 postmortem cases. The femoral blood concentrations of norQTP exceeded those of QTP in four cases. The correlation between QTP and norQTP may be useful in determining the time and route of ingestion [60].

#### 3.2.2. Relationship Between Dose or Concentration and Clinical Presentation

Table 2 shows the characteristics of the 134 patients with QTP intoxication. The median ingested QTP dose for 118 cases was 10 g (IQR 4.25–17.50 g, range 0.25–48 g), and the median c_max_ measured in 65 cases was 4.04 mg/L (IQR 1.83–7.45 mg/L, range 0.03–38.35 mg/L).

**Figure 3:**
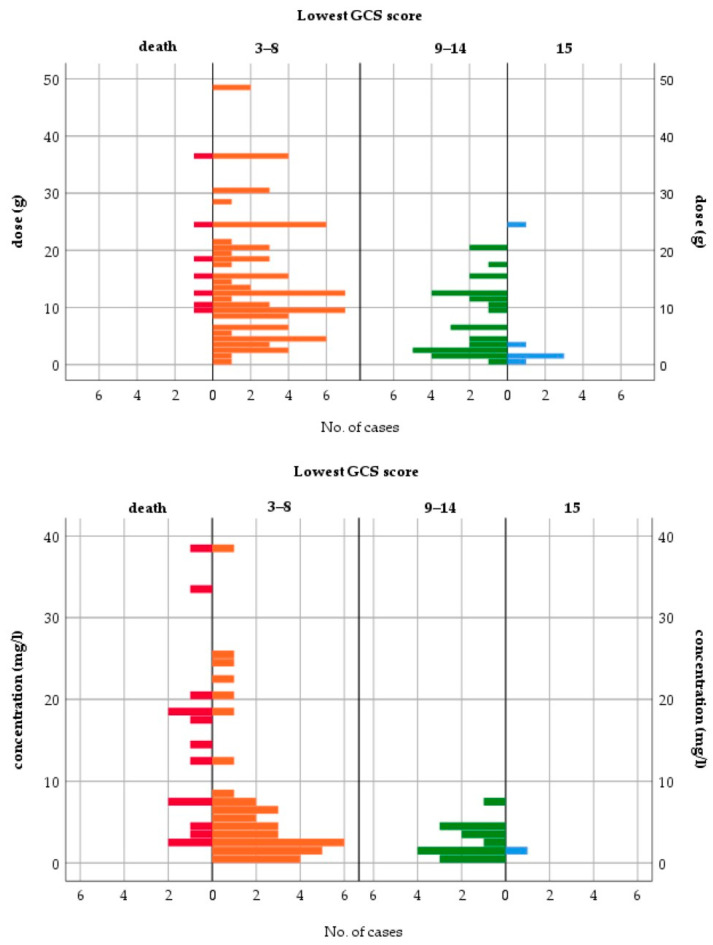
Lowest Glasgow Coma Scale score—case distribution by dose and peak concentration.

All fatalities occurred either with QTP doses ≥ 9 g or c_max_ > 2 mg/L (Figure 3). The median ingested dose and median c_max_ in fatal cases were 15 g and 13.39 mg/L, respectively. CNS depression (GCS < 15) occurred in 95.5% of patients. The case distributions by dose and c_max_ for other toxicodynamic parameters are presented in Supplementary Figures S1 and S2. To assess the relationship between dose or c_max_ and the severity of intoxication, patients with mild to moderate symptoms (GCS ≥ 9) were compared to patients with severe symptoms, including fatal outcomes (GCS ≤ 8). Table 3 shows the results of the logistic regression models for the severity of intoxication.

Dose and c_max_ were good predictors of severe intoxication. Doses > 3 g and c_max_ > 5 mg/L were associated with a higher risk for the lowest GCS score ≤ 8. The previously reported dose thresholds for increasing severity of symptoms were comparable and ranged from 2.5 to 5.4 g [2,97,114,229]. However, individual cases of only mild toxicity at a 20 g dose and survival after a dose of 36 g have been reported [52,220]. For the XR formulation, a dose > 1.5 g and two or more coingested agents were associated with greater severity of poisoning in a retrospective study of 372 cases [26]. Another retrospective analysis of QTP intoxications without sedative coingestants revealed a poor correlation between the ingested dose and the lowest GCS for both formulations. While the signs of peak toxicity were similar for the IR and XR forms, the time to peak toxicity was significantly longer for the XR form: 7 h vs. 3.8 h for the lowest GCS score and 9 h vs. 2.5 h for the maximum heart rate. The median time to recovery from sedation was longer for the XR form (20 h vs. 12 h after IR) [10].

**Figure 4:**
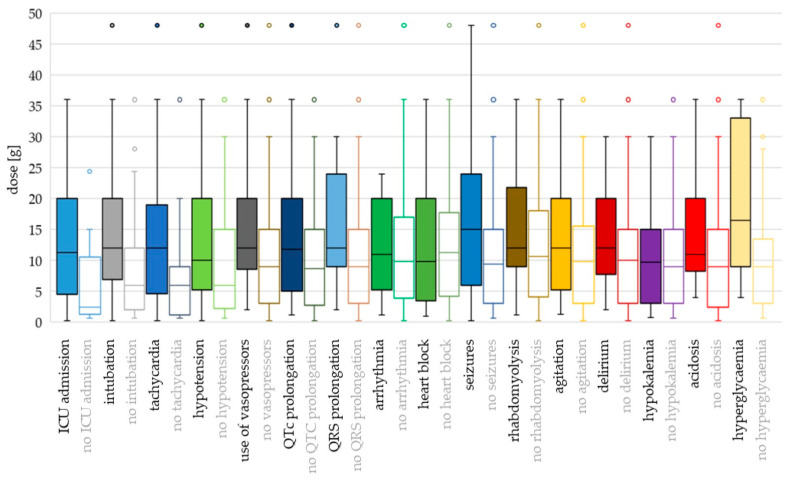
Toxicodynamic parameters vs. ingested quetiapine dose boxplot. Circles denote outlyers by Tukey’s test.

**Figure 5:**
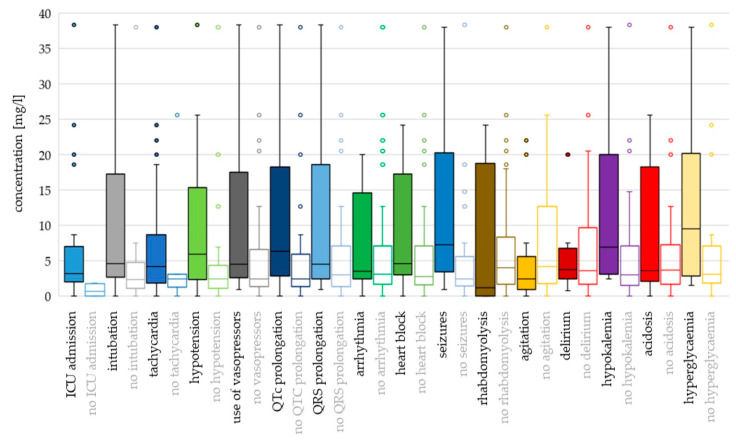
Toxicodynamic parameters vs. quetiapine peak serum concentration boxplot. Circles denote outlyers by Tukey’s test.

For most toxicodynamic parameters, the ingested QTP dose and c_max_ were higher in the “event” groups compared to the “no event” groups (Figure 4 and Figure 5; Supplementary Table S1). Patients without heart block ingested a higher median dose than patients with heart block, and patients without rhabdomyolysis, agitation and acidosis had higher median c_max_ values compared to the “event” groups. However, none of these differences were found to be significant in the logistic regression analyses (Supplementary Table S2).

Hyperglycaemia and seizures occurred at the highest ingested doses and c_max_ values (median dose 16.5 g and 15 g; median c_max_ 9.54 mg/L and 7.30 mg/L, respectively). Median doses were >12 g also for intubation, tachycardia, use of vasopressors, QRS prolongation, rhabdomyolysis, agitation and delirium, while median c_max_ values were >5 mg/L for QT_c_ prolongation, hypotension and hypokalaemia.

Using binary logistic regression with dose as a continuous variable, the differences were significant for ICU admission (OR 1.142; *p* = 0.005), intubation (OR 1.082; *p* = 0.001), tachycardia (OR 1.079; *p* = 0.040), use of vasopressors (OR 1.042; *p* = 0.043), QT_c_ prolongation (OR 1.043; *p* = 0.041) and seizures (OR 1.051; *p* = 0.016). With dose categorisation, the risk of hypotension was higher for doses >8 g compared to doses ≤ 3 g (OR 3.070; *p* = 0.028) (Supplementary Table S2).

C_max_ as a continuous variable was associated with ICU admission (OR 4.263; *p* = 0.023), intubation (OR 1.100; *p* = 0.001) and seizures (OR 1.110; *p* = 0.009). In addition, c_max_ values > 7 mg/L presented greater odds for the use of vasopressors (OR 14.545; *p* = 0.013), values > 4 mg/L were associated with QT_c_ prolongation (OR 4.889; *p* = 0.009) and values >5 mg/L presented greater odds for hypotension, compared to c_max_ ≤ 2 mg/L (OR 7.500; *p* = 0.008).

The odds for ICU admission increased by 14% for every 1 g of QTP ingested and were 6-fold greater for doses between 3 and 8 g and >9-fold greater for doses > 8 g than for doses ≤ 3 g. QTP concentration was a strong predictor of ICU treatment. All patients with a c_max_ > 2 mg/L were admitted to the ICU. In a published case series of 18 patients with QTP poisoning, a dose < 3 g generally did not require ICU admission or hospitalisation for >24 h [2].

The risk of intubation was related to both increasing dose and concentration; the odds were 9.5-fold higher for doses > 4 g QTP (compared to ≤ 2 g), 3.5-fold higher for c_max_ 3-8 mg/L, and 22-fold higher for c_max_ > 8 mg/L (compared to c_max_ < 3 mg/L). In a cohort study of 286 QTP intoxication events, the risk of intubation was dose-dependent, increasing from 10% at a dose of 2 g, 22% at 5 g, 37% at 10 g, and 55% at 20 g [48,106]. The median dose in intubated patients was 5 g (IQR 2.52–11.85 g) [111]. Taylor and Graudins found a higher ingested dose (median 5.8 g) in patients requiring intubation (median 2 g in non-intubated patients). The duration of intubation was significantly longer after ingestion of the XR form compared to the IR form (47 h and 17 h, respectively). However, the ingested doses of the XR form in the study were significantly higher (5.7 g vs. 1.75 g for the IR form) [10].

Apart from CNS depression, tachycardia was the most common sign of QTP intoxication and occurred in >80% of cases. The odds were 12-fold greater for doses 1–10 g and 44-fold greater for doses > 10 g than for doses < 1 g. We also observed a trend of increased risk of tachycardia for c_max_ > 4 mg/L (OR 8.348; *p* = 0.054). There was only one case with c_max_ > 5 mg/L and without tachycardia (compared to 20 cases with tachycardia at c_max_ > 5 mg/L—Supplementary Figure S2).

The risk for hypotension was significantly associated with doses > 8 g (compared to doses ≤ 3 g) and with c_max_ > 5 mg/L (compared to c_max_ ≤ 2 mg/L). Similarly, doses > 7 g demonstrated 4-fold greater odds for the use of vasopressors than doses ≤ 7 g. In a retrospective analysis of 286 QTP intoxication events, a modest correlation was found between the ingested dose and the lowest systolic blood pressure [111].

The lowest dose associated with QT_c_ prolongation was 1.2 g. Increasing dose was significantly correlated with QT_c_ prolongation, confirming previous findings on QT_c_ dose-dependence [76,77,78,79]. The odds for QT_c_ prolongation were 5-fold greater for c_max_ > 4 mg/L. Berling and Isbister analysed the relationship between QTP dose and the risk of uncorrected QT prolongation in 202 patients and found no correlation [230]. Most QT prolongations occurred in patients with tachycardia (rates > 105 bpm) [230]. In our dataset, there was no association between QT_c_ prolongation and tachycardia (Pearson χ^2^(1, N = 103) = 1.88, *p* = 0.170). Uncorrected QT prolongation was not analysed. Several other risk factors for QT_c_ interval prolongation are known, e.g., electrolyte imbalances, female sex, older age, pre-existing cardiac disease and drug–drug interactions [129,132], which were not assessed in this analysis.

The following arrhythmia patterns were described: ventricular tachycardia, ventricular extrasystoles, ventricular fibrillation, self-limiting ventricular bursts, (paroxysmal) supraventricular tachycardia, ectopic atrial rhythm, palpitations, atrioventricular nodal re-entry tachycardia and wide complex rhythm. Arrhythmia was rarely reported, and the number of cases included in analysis was low (seven cases with prolonged QT_c_ interval, four cases without prolonged QT_c_ interval, and three cases with no QT or QT_c_ reported). The association between arrhythmia and QT_c_ prolongation failed to reach statistical significance using Fisher’s exact test (*p* = 0.098). In published studies, acute intoxication with QTP as a single agent did not increase the risk of torsade de pointes [9,111].

There was a small but statistically significant increase of 5% in the odds of seizures for every 1 g of QTP ingested (OR 1.051, *p* = 0.016) and an increase of 10% in the odds for every 1 mg/L increase in c_max_ value (OR 1.110, *p* = 0.009). The odds were 5-fold greater with doses > 20 g (compared to doses ≤ 10 g) and 7.5-fold greater with c_max_ > 4 mg/L. In a cohort study of 286 QTP intoxication events, all five patients who experienced seizures had ingested high doses (6–24 g) of QTP and concurrent drugs, including high doses of citalopram in two cases [111]. In our dataset, seizures occurred at higher dose and concentration ranges than other signs and symptoms, with the exception of hyperglycaemia (median 15 g and 7.3 mg/L, respectively). Half of these patients (17/34) had coingested clinically relevant agents.

There was no association between QTP dose or c_max_ and the risk of QRS prolongation, arrhythmia, heart block, agitation, hypokalaemia or acidosis. There was a trend towards an increased risk of heart block at a c_max_ > 3 mg/L (OR 4.033; *p* = 0.053) and for acidosis at doses > 7 g (OR 3.667; *p* = 0.057). In line with these findings, the occurrence of arrhythmias, AV block, QT_c_ prolongation, hypokalaemia and acidosis in QTP intoxications is not as consistently described as in intoxications with several other antipsychotics [136,231,232]. A recent study by Tang et al. showed no significant increase in QRS prolongation (>120 ms) in QTP poisoning in a large cohort of 11,945 patients with pharmaceutical overdoses [233].

Logistic regression analyses were not performed for rhabdomyolysis, delirium and hyperglycaemia as the number of cases in the “event” group with reported ingested dose or c_max_ was low.

Fourteen cases with confirmed pharmacobezoar formation were included. All involved ingestion of the XR formulation with doses ranging from 3 g to 48 g. None of the cases were fatal. In a series of nine cases of bezoar formation after an overdose of XR QTP, eight patients had coingested other drugs. A gradual or rapid deterioration after several hours was typical in these cases. However, after endoscopic removal of the pharmacobezoars, none of the patients experienced significant toxicity, and all recovered within 3 days [104].

IR and XR forms can differ in their toxicodynamic profile. The IR form may be more likely to cause severe signs, e.g., coma and respiratory depression, in the first hours post-ingestion due to higher c_max_ [29]. On the other hand, symptoms may be prolonged with the XR form, especially if no decontamination has been performed. Fifty-eight of the included patients ingested other clinically relevant agents, most commonly ethanol, benzodiazepines, antidepressants and other antipsychotics, similar to previous reports [26]. Information on the ingested formulation was frequently missing from the reports. It was not feasible to include the QTP formulation, coingested agents or decontamination procedures as covariates in the statistical models with the collected dataset.

In summary, higher QTP dose and concentration were significantly associated with the lowest GCS score, ICU admission, intubation, hypotension, use of vasopressors, QT_c_ prolongation and seizures. Dose, but not concentration, was also associated with tachycardia. The thresholds for increased risk of these events were variable, between 3 g and 20 g for QTP dose and between 2 mg/L and 7 mg/L for QTP concentration. Therefore, doses > 3 g and concentrations > 2 mg/L presented a higher risk of a severe clinical presentation. The probabilities for the parameters, significantly associated with QTP dose and concentration, below and above the 3 g dose and 2 mg/L concentration thresholds are presented in Supplementary Figures S3 and S4.

## 4. Limitations

The reliability of data in the context of intoxication may be questionable. In the existing studies, obtaining accurate information on the dose and time of ingestion presented a challenge, and it was often not documented [1]. In addition, reviewing cases from the literature presents a limitation, as publications may contain different amounts and types of data, and gaining additional information is not possible. Therefore, not all parameters of interest can be captured for every included case. Due to the heterogeneity of data from published reports and studies on acute QTP intoxication, a systematic review was not conducted. However, the literature search, data collection and analysis were clearly structured and described.

With the collected dataset, it was not possible to perform statistical analyses with multiple covariates, as data for different parameters were frequently missing from the references. Imputation, maximum likelihood or a similar method to include missing values was not attempted. Coingestion of other agents was one of the factors that undoubtedly had an impact on the clinical presentation in several cases but was not evaluated.

The frequencies for clinical events, signs and symptoms in the analysis do not represent true incidences, as they are based on published case reports and series and not on population data. The inclusion of published cases has inevitably led to a bias towards more severe cases, as asymptomatic and mild intoxications are not usually published.

The lack of a significant correlation between QTP dose or c_max_ and several toxicodynamic parameters does not mean that these signs and symptoms do not occur in acute QTP intoxication. Rather, it indicates that they may occur regardless of the dose taken or the concentration measured when the QTP dose exceeds the upper therapeutic limit of 800 mg. None of the dynamic parameters were analysed as continuous variables. Therefore, the exact pattern of this relationship cannot be assessed using the data collected from the literature.

## 5. Conclusions

In this structured literature review and analysis of acute QTP intoxication cases, the typical t_1/2_ (16.5 h) was longer than for therapeutic doses. Decontamination procedures may have significantly reduced t_max_, c_max_ and estimated t_1/2_. The ingested QTP dose and c_max_ were significantly correlated. CNS depression (GCS < 15) and tachycardia were the most common signs of QTP intoxication. Dose as a continuous variable was generally a better predictor of clinical events than c_max_. However, with dose and c_max_ categorisation, both were significantly correlated with the same clinical events. Higher doses and c_max_ values increased the risk of ICU admission, intubation, hypotension, use of vasopressors, QT_c_ prolongation and seizures, but not QRS prolongation, arrhythmia, heart block, agitation, hypokalaemia or acidosis. Tachycardia was associated with increasing doses and was short of a significant correlation with c_max_. The dose thresholds for an increased risk of “event” for individual toxicodynamic parameters were typically between 3 g and 7 g (20 g for seizures and QT_c_ prolongation), and the c_max_ thresholds were between 2 mg/L and 4 mg/L (7 mg/L for the use of vasopressors). Therefore, doses > 3 g and c_max_ > 2 mg/L may be considered alert levels for high risk of severe complications in acute QTP intoxication.

Patients ingesting >3 g of QTP should be observed at the Emergency Department and may be transferred to a psychiatric unit no sooner than after 6 h if no severe signs and symptoms are present. The type of formulation (IR or XR) should be specified together with the dose. If ingestion of XR QTP is suspected, patients may require longer, e.g., 12 h observation. Prompt decontamination (e.g., use of activated charcoal within 6 h post-ingestion) is likely to improve the clinical course of intoxication. If a serum QTP concentration measurement is available on site, sampling upon admission, but not earlier than 2 h post-ingestion, is recommended to confirm or exclude the intoxication with QTP. A concentration > 2 mg/L indicates a severe, potentially lethal intoxication. However, lower concentrations may still anticipate a severe clinical course, depending on the time from ingestion to sampling, so caution is required when interpreting concentration results.

## 6. Future Directions

Future studies may investigate closely the duration of common signs and symptoms, particularly when evaluating treatment strategies. The reports on QTP intoxication should state the formulation (IR or XR) that may influence the clinical course and is relevant for clinical decision-making. Determining the concentration–time relationship for two analytes, QTP and its active metabolite norQTP, could be informative. The measurement of both analytes in clinical practice might improve the estimation of the time from ingestion to sampling.

## Figures and Tables

**Table 1:** Pharmacokinetic and toxicokinetic parameters of quetiapine [2,10,19,27,28,32,33,34,35,36,37,39,40,41,42,43,44,45,46,47,48,49,50,51,52,53,54,55,56,57,58].

	Pharmacokinetic Profile		Toxicokinetic Profile		
	IR	XR	IR	XR	Comments
bioavailability	70%	equivalent to IR	variable and lower than in therapeutic doses; e.g., 40% after 10 g IR	no data—lower than in therapeutic doses	
k_a_	1.77–2.5/h	0.1–0.19/h	2.3/h	no data—lower than IR	gastric emptying may be delayed
V_D_	380–710 L, or 8–14 L/kg	equivalent to IR	589 L	equivalent to IR	
t_max_	1–2 h	5–6 h	variable, 1–20 h	variable, up to 56 h	c_max_ may not have been reached at the reported time
c_max_	dose-dependent, with linear pharmacokinetics	dose-dependent, with linear pharmacokinetics, lower (~67%) than IR	conflicting data on dose–concentration relationship; lower than anticipated from pharmacokinetic models		2nd peak may be present
t_1/2_	5.3–7 h	equivalent to IR	variable, 5–22 h; may be biphasic, with phase 1 t_1/2_ 5–10 h (<12–48 h post-ingestion, and phase 2 t_1/2_ 22–77 h (>12–48 h post-ingestion)	variable; may be prolonged—up to 31 h	
Cl	55–138 L/h	equivalent to IR	73 L/h	no data	declines with age

c_max_: peak concentration; Cl: apparent clearance; IR: immediate-release; k_a_: absorption rate constant; t_max_: time to peak concentration; t_1/2_: half-life; V_D_: volume of distribution; XR: extended-release.

**Table 2:** Patient demographics, clinical presentation and treatment strategies.

				Reported QTP Dose	Reported QTP Concentration
		No. of Cases * (N = 134)	% (Valid)	No. of Cases * (N = 118)	No. of Cases * (N = 65)
age—median (IQR)	years	32.5 (24.75–44)			
sex	male/female	55/76	(41.9/58.1)	49/66	23/41
coingestants	yes/no	58/76	(43.3/56.7)	49/69	28/37
dosage form	IR/XR	53/30	(63.9/36.1)	49/28	31/13
hospital length of stay—median (IQR)	days	4 (2–7)			
Clinical events, signs and symptoms:					
lowest GCS score level	1 (death)	17	12.8	7	14
	2 (GCS 3–8)	78	58.6	74	36
	3 (GCS 9–14)	32	24.1	30	14
	4 (GCS 15)	6	4.5	6	1
tachycardia	yes/no	93/21	(81.6/18.4)	81/19	47/9
hypotension	yes/no	65/44	(59.6/40.4)	54/41	34/21
QT_c_ prolongation	yes/no	44/67	(39.6/60.4)	40/61	21/31
QRS prolongation	yes/no	24/90	(21.1/78.9)	19/83	15/39
seizures	yes/no	34/89	(27.6/72.4)	27/82	21/37
rhabdomyolysis	yes/no	12/90	(11.8/88.2)	12/80	4/40
agitation	yes/no	32/87	(26.9/73.1)	28/78	21/35
delirium	yes/no	17/92	(15.6/84.4)	13/83	12/38
hypokalaemia	yes/no	17/66	(24.1/75.9)	15/59	7/35
acidosis	yes/no	19/79	(19.4/80.6)	16/72	13/36
arrhythmia	yes/no	14/104	(11.9/88.1)	10/98	8/47
heart block	yes/no	21/97	(17.8/82.2)	16/92	14/42
hyperglycaemia	yes/no	9/70	(11.4/88.6)	8/64	7/32
Treatment strategies:					
decontamination (any)	yes/no	58/58	(50.0/50.0)	55/51	27/26
ICU admission	yes/no	66/20	(76.7/23.3)	61/20	32/6
ICU length of stay—median (IQR)	days	3 (1–4)			
intubation	yes/no	72/55	(56.7/43.3)	64/29	34/27
physostigmine	yes/no	14/111	(11.2/88.8)	12/100	8/51
vasopressors	yes/no	35/87	(28.7/71.3)	29/79	21/38
intralipid emulsion	yes/no	18/107	(14.4/85.6)	17/95	7/52

GCS: Glasgow Coma Scale; ICU: Intensive Care Unit; QTP: quetiapine; * For each parameter, cases with missing data are not presented.

**Table 3:** Lowest Glasgow Coma Scale (≤8 or ≥9) vs. quetiapine dose and peak concentration—logistic regression results.

			OR	95% C.I. for OR		Sig.	Omnibus Test—Model (Sig.)	Hosmer and Lemeshow Test (Sig.)
				Lower	Upper			
lowest GCS score vs. dose (continuous)		dose (g)	1.118	1.049	1.192	0.001	<0.001	0.149
		constant	0.711			0.334		
lowest GCS score vs. dose (categorical; cat. 1: dose ≤ 3 g; cat. 2: 3 g <dose ≤ 8 g; cat. 3: dose > 8 g)		dose ≤ 3 g (ref.)				0.000	<0.001	1.000
		3 g <dose ≤ 8 g	5.950	1.586	22.328	0.008		
		dose > 8 g	8.955	3.221	24.899	0.000		
		constant	0.471			0.079		
lowest GCS score vs. peak concentration (continuous)		peak c (mg/L)	1.319	1.020	1.707	0.035	0.001	0.974
		constant	1.015			0.975		
lowest GCS score vs. peak concentration (categorical; cat. 1: peak c ≤ 5 mg/L; cat. 2: peak c > 5 mg/L)		peak c > 5 mg/L	14.000	1.709	114.685	0.014	0.001	*
		constant	1.786			0.082		

C.I.: confidence interval; GCS: Glascow Coma Scale; OR: odds ratio; * Hosmer and Lemeshow test not calculated for dichotomous independent variable.

## Data Availability

No new data were created in this study. Statistical analyses are provided as Supplementary Materials. A data spreadsheet with collected information on the cases included in the analysis is available upon request from the authors.

## Supplementary Materials

The following supporting information can be downloaded at: https://www.mdpi.com/article/10.3390/jox14040085/s1, Figure S1: Toxicodynamic parameters—case distribution by dose; Figure S2: Toxicodynamic parameters—case distribution by peak concentration; Figure S3: Toxicodynamic parameters—probabilities with quetiapine dose below and above 3 g; Figure S4: Toxicodynamic parameters—probabilities with quetiapine concentration below and above 2 mg/l; Table S1: Dose and peak concentration data for toxicodynamic parameters; Table S2: Toxicodynamic parameters—logistic regression results.
