# Supplementary figures and images for: Acute Quetiapine Intoxication: Relationship Between Ingested Dose, Serum Concentration and Clinical Presentation—Structured Literature Review and Analysis
Source: J Xenobiot. 2024 Oct 18;14(4):1570–94. doi: 10.3390/jox14040085 (PMC11503392; doi:10.3390/jox14040085)

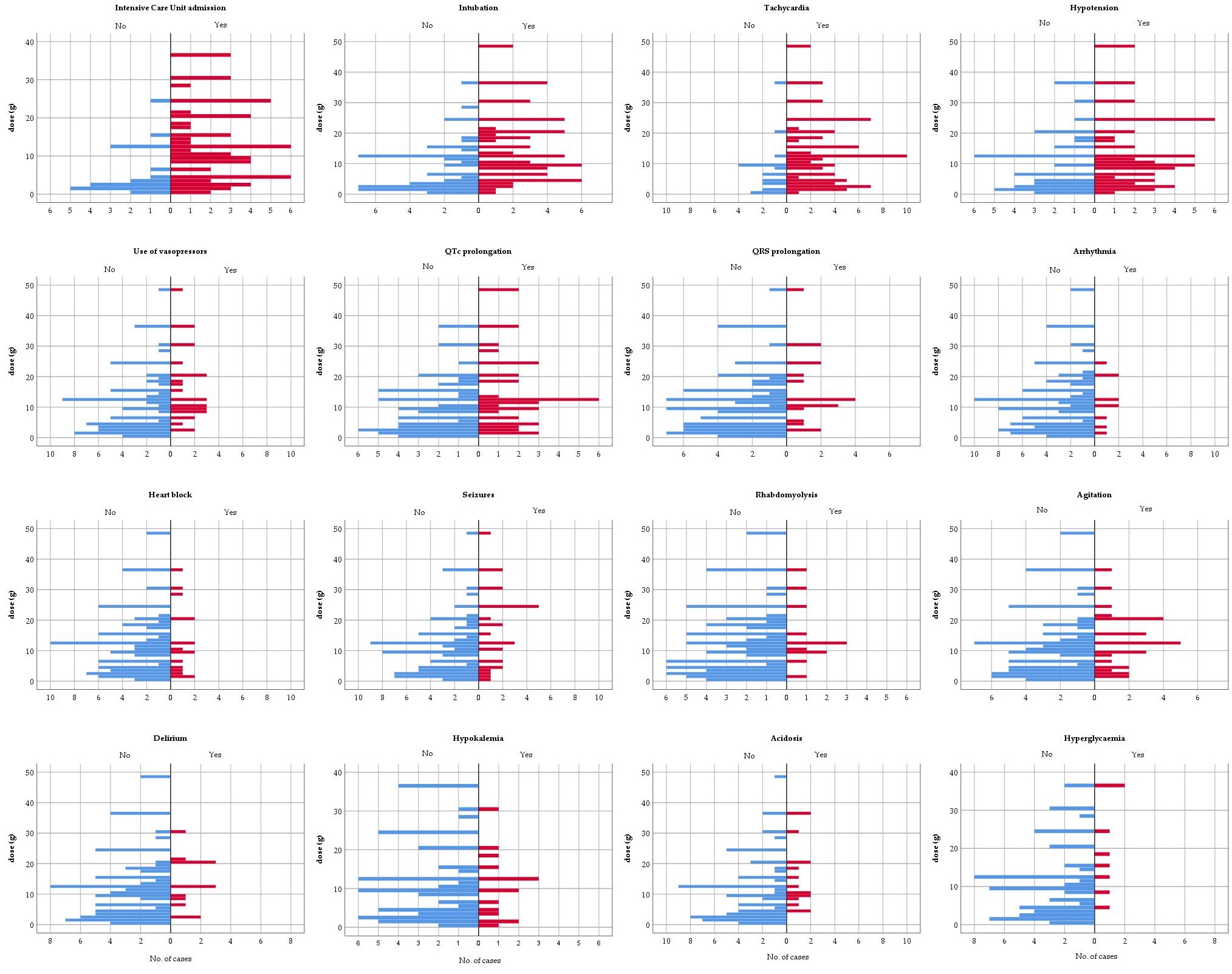

Supplement: Supplementary file 1 [file jox-14-00085-s001.zip › Supplementary Figure S1. Toxicodynamic parameters - case distribution by dose.jpg]

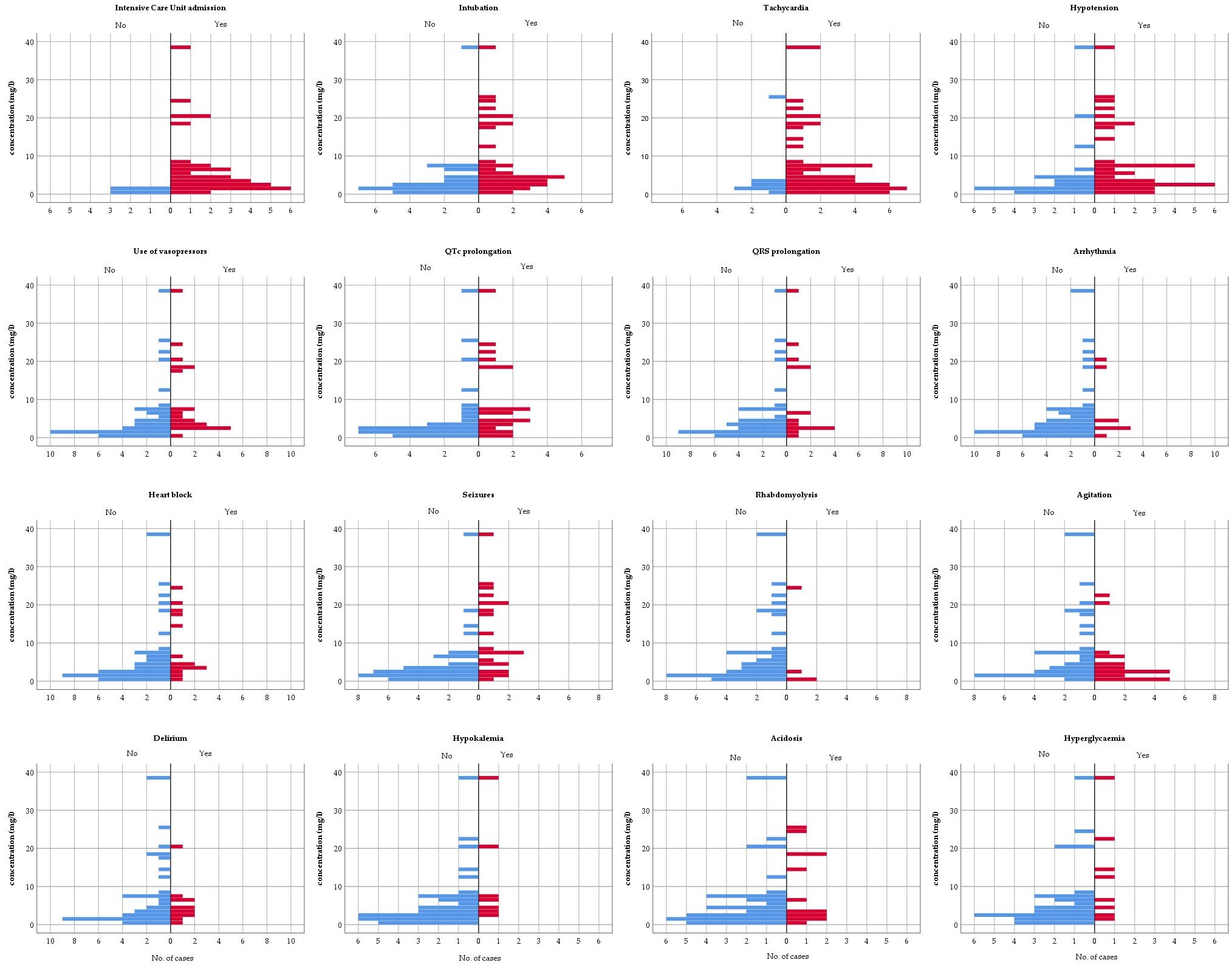

Supplement: Supplementary file 1 [file jox-14-00085-s001.zip › Supplementary Figure S2. Toxicodynamic parameters - case distribution by concentration.jpg]

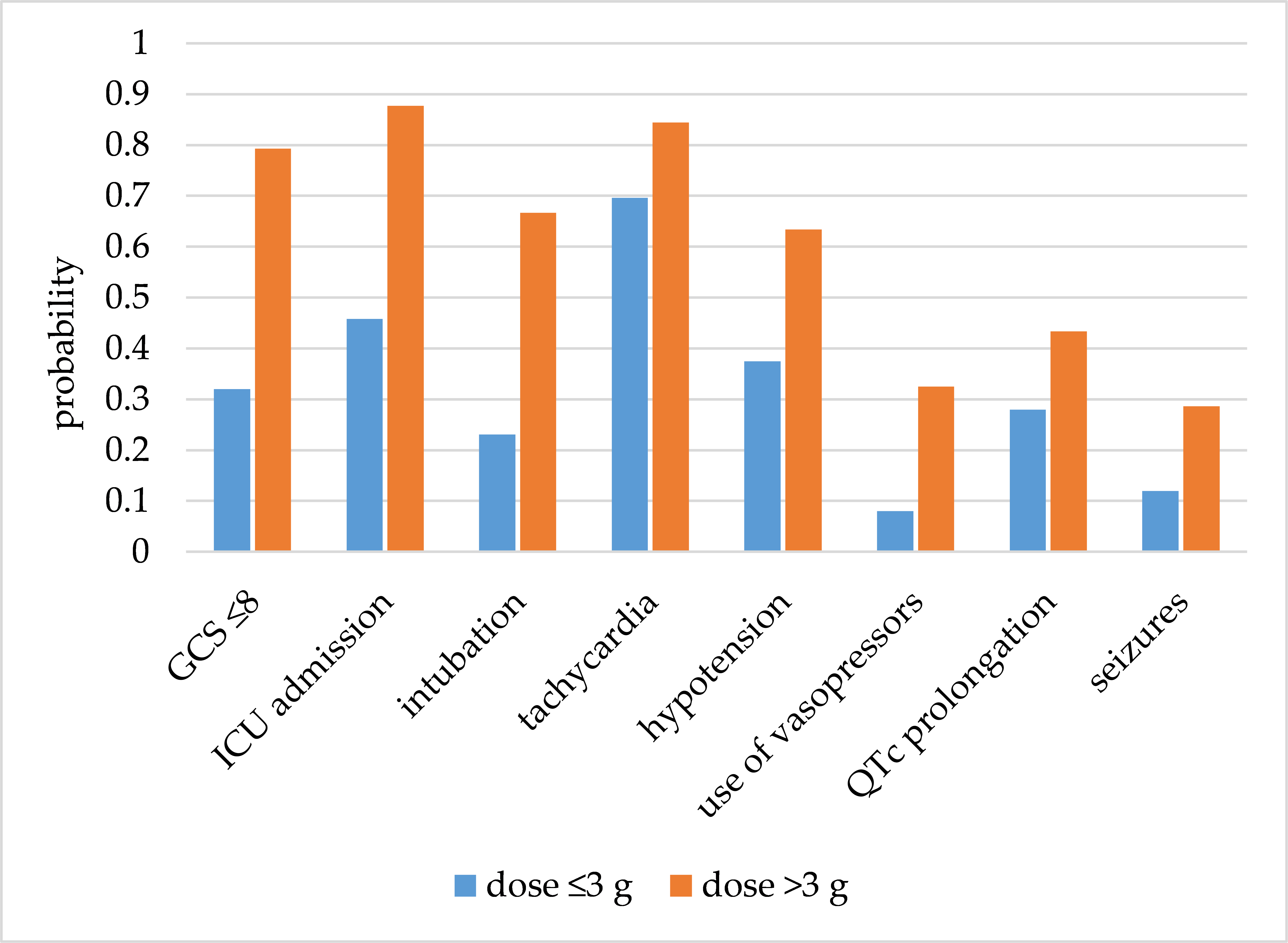

Supplement: Supplementary file 1 [file jox-14-00085-s001.zip › Supplementary Figure S3. Toxicodynamic parameters - Probabilities with quetiapine dose below and above 3 g.png]

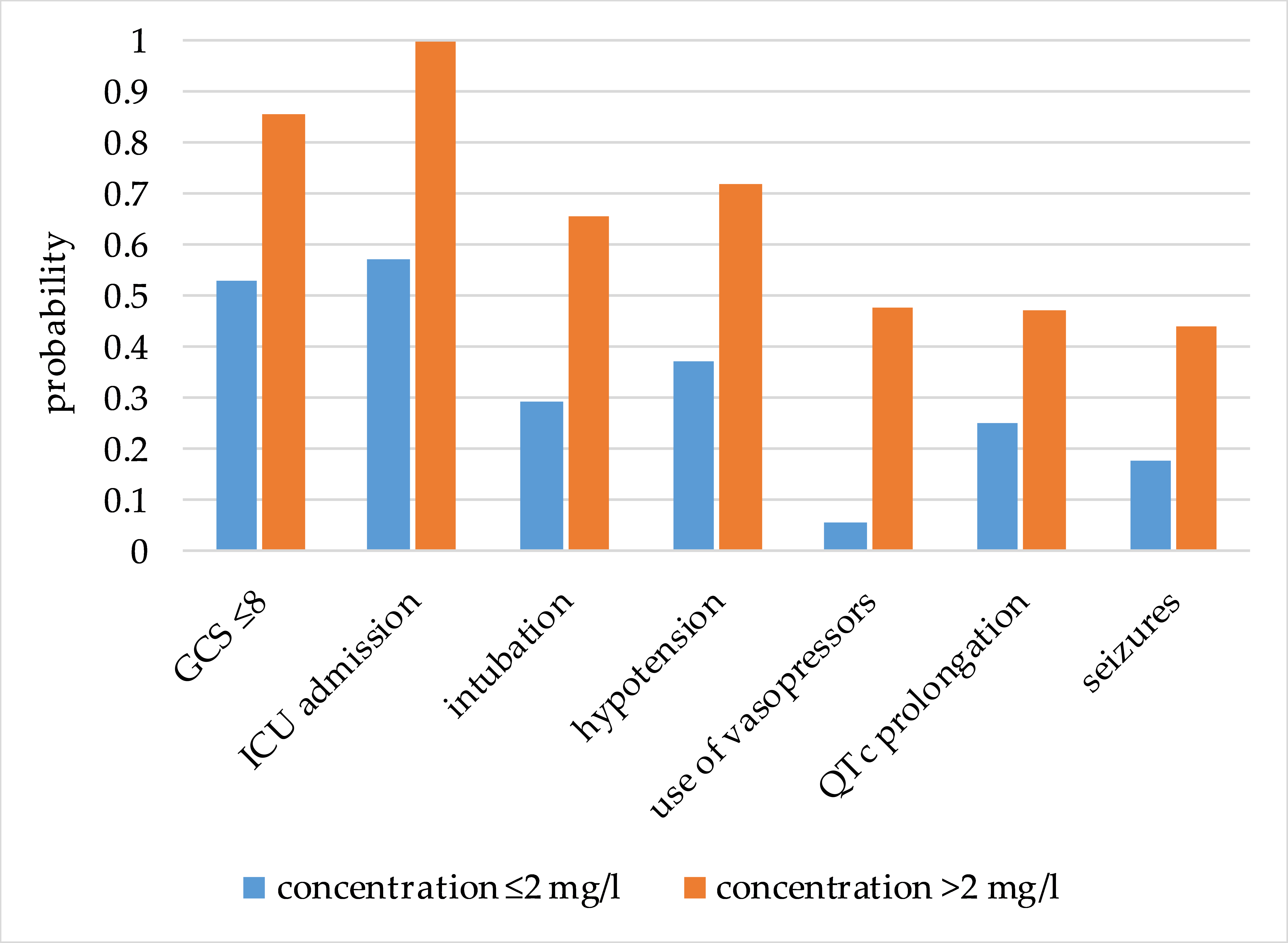

Supplement: Supplementary file 1 [file jox-14-00085-s001.zip › Supplementary Figure S4. Toxicodynamic parameters - Probabilities with quetiapine concentration below and above 2 mgl.png]
